# Dissecting the genetic basis of rice husk silica content and morphology using GWAS

**DOI:** 10.1007/s00425-026-05211-8

**Published:** 2026-10-22

**Authors:** Masoumeh Kordi, Forozan Jalali Bidgoli, Naser Farrokhi, Pär K. Ingvarsson, Asadollah Ahmadikhah

**Affiliations:** 1 Department of Cell & Molecular Biology, Faculty of Life Sciences & Biotechnology, Shahid Beheshti University, Tehran, Iran; 2 Department of Plant Biology, Swedish University of Agricultural Sciences, Uppsala, Sweden

**Keywords:** Agricultural waste, Bioeconomy, Epistatic interaction, Haplotype analysis, Linkage disequilibrium, *Oryza sativa*, QTL, SNP–SNP interactions

## Abstract

**Main conclusion:**

Our findings reveal a complex genetic architecture underlying rice husk silica traits, identifying key QTLs, candidate genes, and epistatic interactions with potential applications in rice breeding and silica valorization.

**Abstract:**

Rice contains large amounts of amorphous silica in its tissues. Rice husk (RH) contains 17–30% silica that can be recycled for use in a variety of industries. A genome-wide association study (GWAS) was performed to pinpoint the genetic determinants of five RH features: silica content, silica block length and width, thorn (cone-shaped protrusions on the surface of the husk) height, and base length. A data set of 44,100 SNPs from rice genotypes was used for genomic analysis. In total, 48 significant SNPs were identified, corresponding to 23 unique QTLs across nine rice chromosomes. The most significant SNPs and QTLs associated with silica traits are located on chromosomes 2, 6, and 9. The candidate genes in the definition of RH silica-related traits are involved in growth and development (zinc finger protein, esterase SGNH hydrolase-type domain-containing protein, glycosyltransferase), heavy metal detoxifying and transporting (ABC transporter, BTB domain-containing protein, heavy metal transport/detoxification protein domain-containing protein, ankyrin repeat domain-containing), stress response (genes coding for chitinase, peroxidases, polyphenoloxidases, β-1,3-glucanase, phenylalanine ammonia-lyase, superoxide dismutase, ascorbate peroxidase, glutathione reductase, catalase, lipoxygenase, glucanase, F-box protein, NB-ARC domain-containing protein, thylakoid membrane phosphoprotein), and transcription regulation (MYB transcription factor, TCP domain-containing protein). Haplotype analysis identified haplogroups qSBL91 and qTH31 for two traits of SBL (length of silica blocks) and TH (height of the silica thorns). An analysis of epistatic interactions among candidate SNPs identified 18 significant SNP-SNP interactions between cytochrome P450, glycosyl hydrolases, laccases, zinc finger, and bHLH.

**Supplementary Information:**

The online version contains supplementary material available at https://doi.org/10.1007/s00425-026-05211-8.

## Introduction

Silica is found in various natural sources, from rocks such as granite, sedimentary rocks, marble, raw minerals such as quartz and gypsum, and volcanic glass (Raju and Rom 1998; https://www.osha.gov/silica-crystalline), to biogenic silica, including plant waste such as rice and wheat husks, corn cob and sugarcane bagasse, and living organisms such as diatoms (Thakur et al. 2024). Silicon positively affects plant health by reducing abiotic and biotic stresses and increasing plant resistance to pests and pathogens (Islam et al. 2023). The beneficial effects of silica on plant growth are attributed to improved mechanical strength and the outer protective layer, activation of defense-related enzymes, stimulation of antimicrobial compound production, regulation of complex signal pathways, and activation of defense-related gene expression (Wang et al. 2017a, b). Husk is a somewhat neglected main agricultural waste/by-product in rice-growing countries. Rice husk (RH) is made of hollow fibers of cellulose (25–33%), lignin (25–31%), hemicellulose (18–21%), pentosans (15%) and minerals such as silica (16%) (Gao et al. 2018; Chen et al. 2020; Katz et al. 2021; Prabha et al. 2021). Silica is amorphous in plants as compared to the crystalline form found in soil. This heterogeneity allows industrially important chemical reactions to occur (Al Soubaihi et al. 2020). Rice-derived silica has been used in various industries, including glass ceramics (Aremo et al. 2024), refractory and aerogel composites (Oli and Sharma 2026), concrete (Berktas et al. 2021; Farid and Zaheer 2023), brick (Hossain et al. 2021; Kumar et al. 2025), pharmacy and medicine (Ismail et al. 2021), drug delivery (Nzereogu et al. 2023), energy (Nzereogu et al. 2023), absorption (Nzereogu et al. 2023), catalysis (Nzereogu et al. 2023), and fertilizer and pesticide formulation (Kordi et al. 2025a). Rice silica offers an environmentally friendly, cost-effective alternative for sustainable resource use, improved waste management, and reduced environmental pollution.

**Fig. 1:**
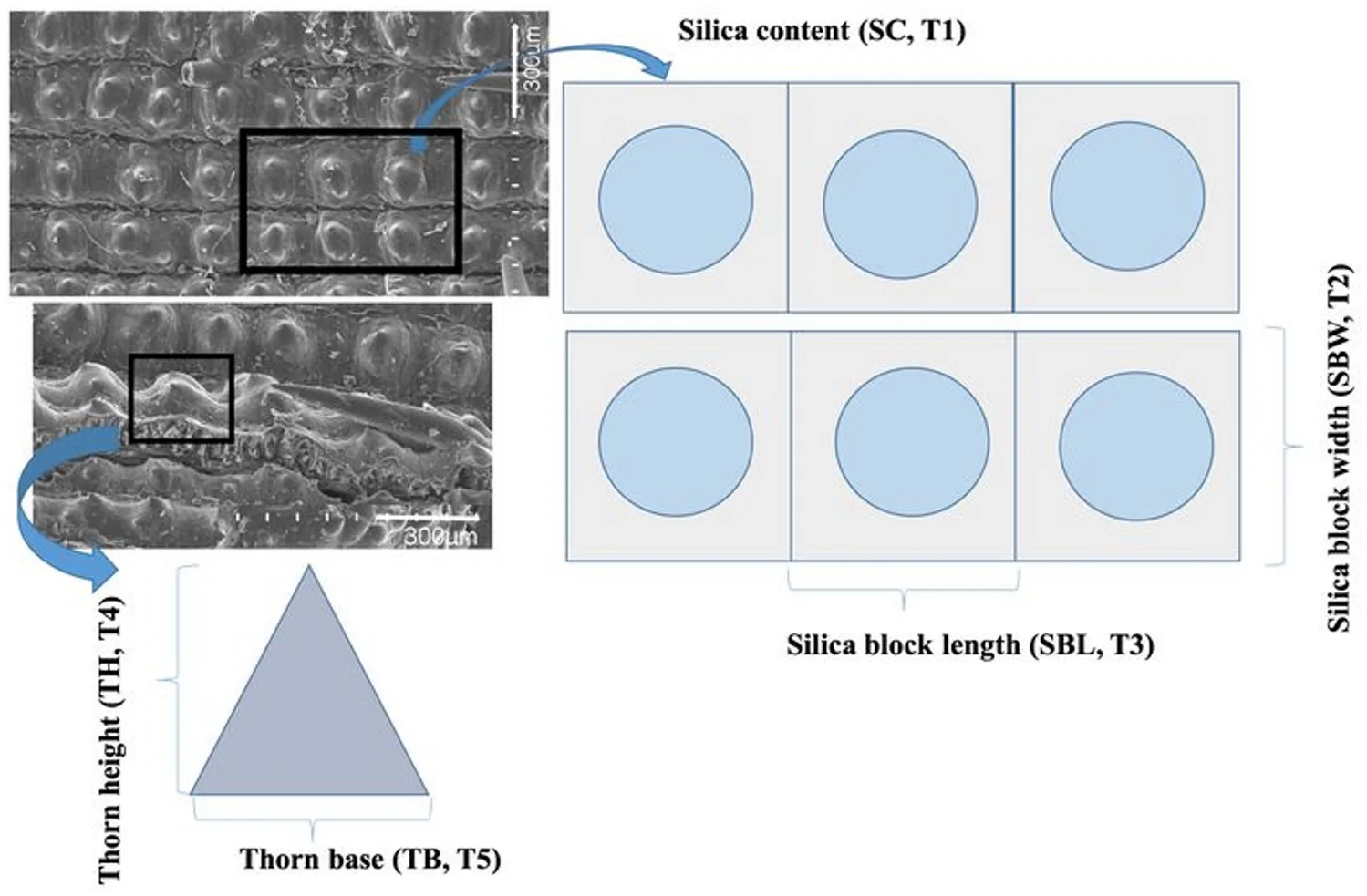
Rice husk (RH) corresponding traits at the microscopic scale (300 µm). Silica block width (SBW, T1), silica block length (SBL, T2), thorn height (TH, T3), thorn base (TB, T4), and silica content (SC, T5) are shown (15.0 kV, 150 SE, 300 µm)

In the search for QTLs and corresponding genes involved in rice silica content, 258 *Oryza nivara* were checked for straw silica content (Gowda et al. 2023). Marker-trait associations (*n* = 14) were identified, yielding a few candidates, including ATP-binding cassette (ABC) transporters, Casparian thickening, multi-drug and toxin extrusion (MATE) proteins, F-box, and MYB transcription factors (Gowda et al. 2023). Here, for the first time, we dissected RH silica content and arrangements using a GWAS mapping population of 153 rice genotypes. QTLs for five traits (Table 1, Fig. 1) were identified. These features quantify the apparent differences between RH accessions. Our findings provide the basis for detailed molecular analyses of RH silica features.

**Table 1:** List of trait names, abbreviations (symbol), descriptions, and units (Fig. 1)

Trait name	Symbol	Trait description	Measurement unit
Silica content	SC	The silica percentage in each sample	Percentage
Silica block width	SBW	The width of silica blocks	Pixel
Silica block length	SBL	The length of silica blocks	Pixel
Thorn height	TH	The height of the silica thorns	Pixel
Thorn base	TB	The base of silica thorns	Pixel

## Materials and methods

### Plant materials and growing conditions

We used 283 rice accessions (*Oryza sativa* L.), including landraces, breeding lines, varieties, and cultivars from 82 countries, plus three local cultivars as check varieties (Sadri, Sang Tarom, Dom siah Kalat). Accessions belonged to TEJ (temperate japonica), IND (indica), AUS (aus), ARO (aromatic), TRJ (tropical japonica) ADMIX subpopulations. Out of 283 accessions, 153 reached full maturity during the growing season (2023–2024) in an augmented block design with 3 replicates in an experimental field at Sari Agricultural University, Iran. Field-harvested accessions (153) were fully mature with ripe panicles. We applied standard field management practices throughout the growing season. All panicles were collected only from the main tiller, i.e., the tallest panicle, at maturity. The seeds were harvested and transported to the laboratory (Table S1).

### Silica extraction method

RH samples (10 g) were separated with a scalpel from seeds in each accession of *O*. *sativa*. We used an acid leaching method, with some modifications, for silica extraction (Azat et al. 2019). Briefly, RH was washed sequentially with dH_2_O, HCl (1 N) for 1 h at 100 °C, and 6× dH_2_O. The washed samples were incubated at 70 °C for 24 h and then at 600 °C for 6 h. For each genotype, husk silica percentage was calculated as the ratio of ash weight to initial weight. To perform surface imaging of rice husks from various genotypes by, the samples were mounted on a holder. They were gold-sputtered and imaged using a scanning electron microscope (SEM).

### Morphological characteristics of silica in burnt husk

**Fig. 2:**
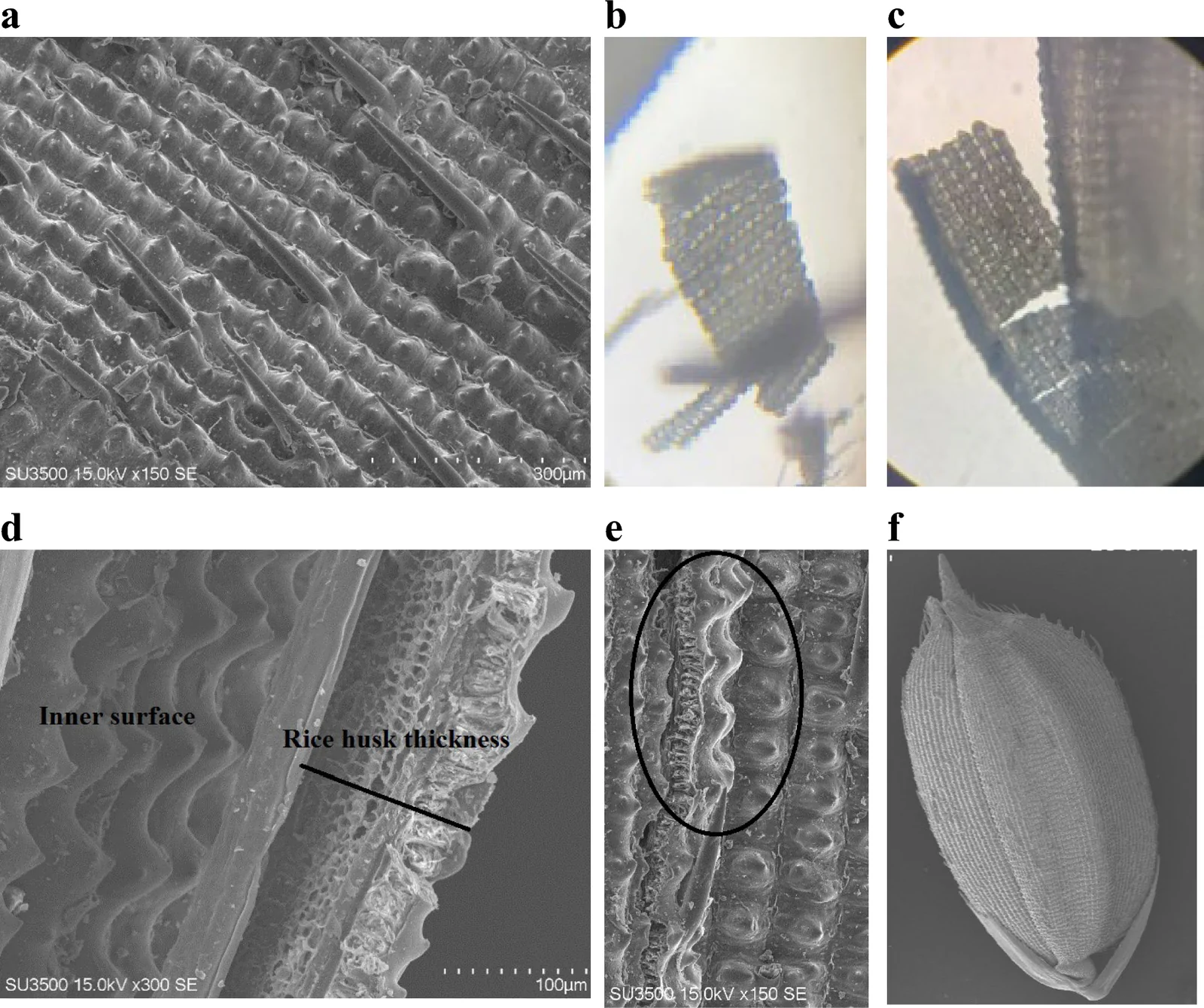
**a** Scanning electron microscopy (SEM) from the outer layer of RH accessions. **b**, **c** Light microscope images of RH after burning. **d-f** SEM of RH thickness and cross-section width of the cells forming the husk. View of the inner surface of the rice husk (**d**), thorn size shown in the circle (**e**), and outer surface of RH-coated rice (**f**)

A sample of burnt RH from all genotypes was placed on a slide and images were taken under a light microscope at 40× magnification. The images were analyzed using Tracker 6.1.6 software, and the traits (SC, SBW, SBL, TH and TB, Figs. 1, 2) were measured.

### Genome-wide association studies

Zhao et al. (2011) previously described SNP sequencing and array hybridization. Single-nucleotide polymorphism (SNP) information for the studied genotypes was downloaded from the Gramene portal (http://gramene.org). We used 44,100 SNPs from two data sources, i.e. the *Oryza* SNP project and OMAP project, for genome-wide association studies (GWAS). Minor allele frequency (MAF) was considered less than 0.05 and removed from the dataset. PCA was used for population structure analysis with the rMVP package (https://cran.r-project.org/web/packages/rMVP/index.html). GWAS analysis was performed using mixed linear models (MLM) (Zhang et al. 2010), the fixed and random model circulating probability unification (FarmCPU), and the general linear model (GLM) in rMVP (Liu et al. 2016; Panahabadi et al. 2021; Kordi et al. 2025b). The Manhattan plots were generated using –log_10_(*p*) values using the MVP packages in R (Panahabadi et al. 2021; Kordi et al. 2025a). We considered SNP markers with −log_10_(*p*-value) > 3.7 and 4 significant, based on visual inspection of the quantile–quantile (Q–Q) plots. A phylogenetic tree and heatmap of the values in the kinship (k) matrix were created from the numeric SNP data by the Popkin package in R (Panahabadi et al. 2021; Kordi et al. 2025c). Linkage disequilibrium decay was estimated among all 153 accessions in the association panel. The resolution of LDs was evaluated by pairwise calculations of LDs between 34,072 SNP markers. The r^2^ value in a sliding window of 50 markers was used in TASSEL and plotted graphically using ggplot2 in R. The plots depicted LD decay with the physical distance between the SNPs (Luo et al. 2019). Based on the LD decay of each chromosome, the left and right r^2^ half-decay regions flanking each SNP were determined to identify candidate genes (Luo et al. 2019; Kordi et al. 2025b). We shortlisted candidate genes based on annotated function and conducted a comprehensive literature review to investigate associations between candidate genes surrounding QTLs and silica-related traits. Genes with pleiotropic effects between traits were also identified.

### Haplotype and epistatic analyses

Haplotype analysis was performed for all gene blocks using the GeneHapR package in R. Haplogroups consisting of fewer than five accessions were excluded. To determine potential genic interactions between different QTLs influencing RH silica traits, epistatic interaction analysis was performed using functional regression with FRGEpistasis in R.

## Results

### Population structure and LD decay

RH silica content ranged from 3.53 to 35.45% (highest: g248; lowest: g133) among the 153 accessions (Table S2). RH ash showed different morphologies (Table S2). Phenotypic distributions for all traits are shown in Fig. S1 and represented in Table S2. Principal component analysis (PCA) revealed a distinction among the subpopulations (Fig. S2), viz. ADMIX, AROMATIC, AUS, IND, TEJ, TRJ according to Zhao et al. (2011), grouping them into four classes, which were different from our classification according to PCA (three classes, Fig. S2). A total of 34,072 high-quality SNPs were distributed uniformly across rice chromosomes, based on MAF calculations. The genetic relationships among the accessions were assessed by generating a kinship matrix derived from the filtered SNPs (Fig. S3). The results showed five subpopulations of temperate *japonica*, indica, Aus, aromatic, and tropical japonica (Fig. S3). LD decay for all chromosomes is presented in Fig. S4. The mean values of LD based on half r^2^ values varied from 170 kbp (for chromosome 11) to 875 kbp (for chromosome 7) across the rice genome (Table S3). The distribution of SNP markers on each chromosome is provided in Fig. S5. The most and the least abundant were on chromosomes 7 and 11, respectively. On average, a SNP was observed every 10,114 bp in the rice genome (Table S3).

### Genome-wide association studies

**Fig. 3:**
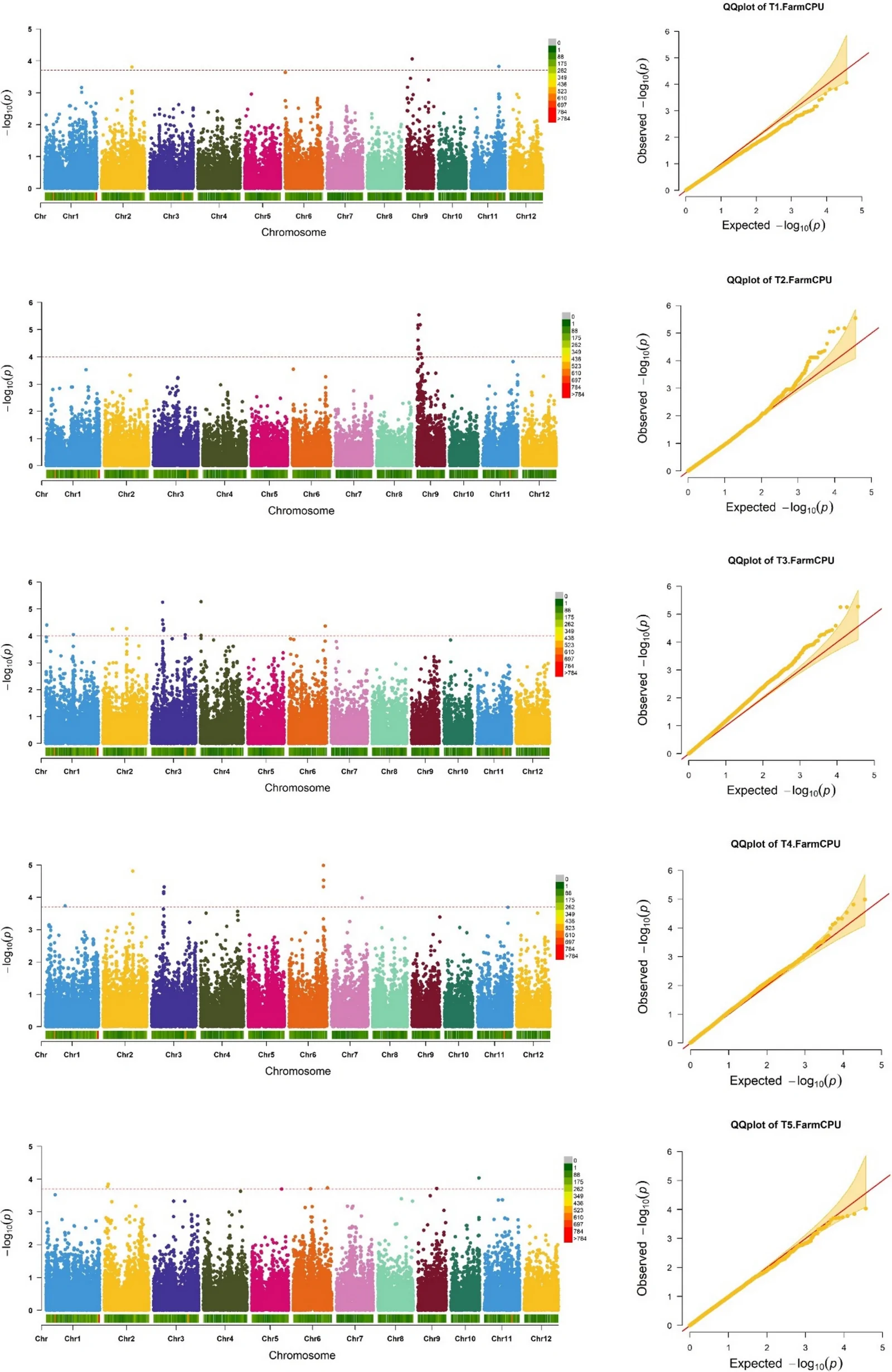
Manhattan plots and Q-Q plots for all traits: Silica block width (SBW, T1), silica block length (SBL, T2), thorn height (TH, T3), thorn base (TB, T4), silica content (SC, T5)

In this study, 34,072 SNPs were used for association mapping. According to the Manhattan plots (Fig. 3), 48 SNPs with −log_10_(*p*) values ≥ 3.7 and had an association with five traits. Significant SNPs were distributed on all chromosomes except for chromosomes 5, 8 and 12 (Table S4). The candidate genes were found in 23 QTL regions with significant SNPs (Table S5). Traits with the shared SNPs are listed in Table S6. The peak markers, −log10(*p*), candidate genes, QTLs, and candidate gene IDs are presented in Table S7. The candidate gene functions are presented in Table S8. According to comparative Q–Q plot analyses between GWAS models (GLM, MLM, and FarmCPU) for all traits, the FarmCPU model was chosen because it detected more marker-trait associations (Fig. 3). Pleiotropy occurs when one gene affects more than one trait and contributes to the genetic correlation between traits (Auge et al. 2019). Pleiotropy, together with LD, causes genetic correlation between traits due to physical linkage or population structure (Chebib and Guillaume 2021). Genes with pleiotropic effects with potential functions in several traits are reported in Table S8. These genes include leucine-rich repeat genes, similar to laccase 90c, fructose−6-phosphate−2-kinase/fructose−2,6-bisphosphatase, and serine/threonine-protein kinase, among others (Table S9).

### Candidate gene mining in QTL regions

Several candidate genes were found in the vicinity of GWAS signals for different traits. Overall, some of the candidate genes were also reported on silica content in other crops. They were coding for an ABC transporter (Song et al. 2014; Hinrichs et al. 2017), an F-box protein (Frouin et al. 2019; Gowda et al. 2023), and a MYB transcription factor (Xu et al. 2023; Roy et al. 2024). Reported for the first time in relevance to RH silica (Table S7) are laccase 90c, lipoxygenase, esterase SGNH hydrolase type domain containing protein, PEPC kinase, leucine-rich repeat, homeodomain-like containing protein, glycosyltransferase, phenylalanine ammonia-lyase, NB-ARC domain containing protein, heavy metal transport/detoxification protein domain containing protein, disease resistance protein domain containing protein, ankyrin repeat domain containing, DAG protein, lipase GDSL domain containing and thylakoid membrane phosphoprotein.

### Haplotype analysis

Haplotype analysis of SBL (silica block length) showed two haplotypes at the qSBL91 region on chromosome 9 [H001 (present in 105 accessions with wide distribution around the world, more in Europe and Asia) and H002 (present in 32 accessions originating from America and Africa, Australia and South Asia)] with no significant difference between the two, as illustrated by a violin plot. H001 mostly contains TEJ, IND, AUS and ADMIX, while H002 mostly contains accessions from TRJ, ADMIX and TEJ (Fig. S6).

**Fig. 4:**
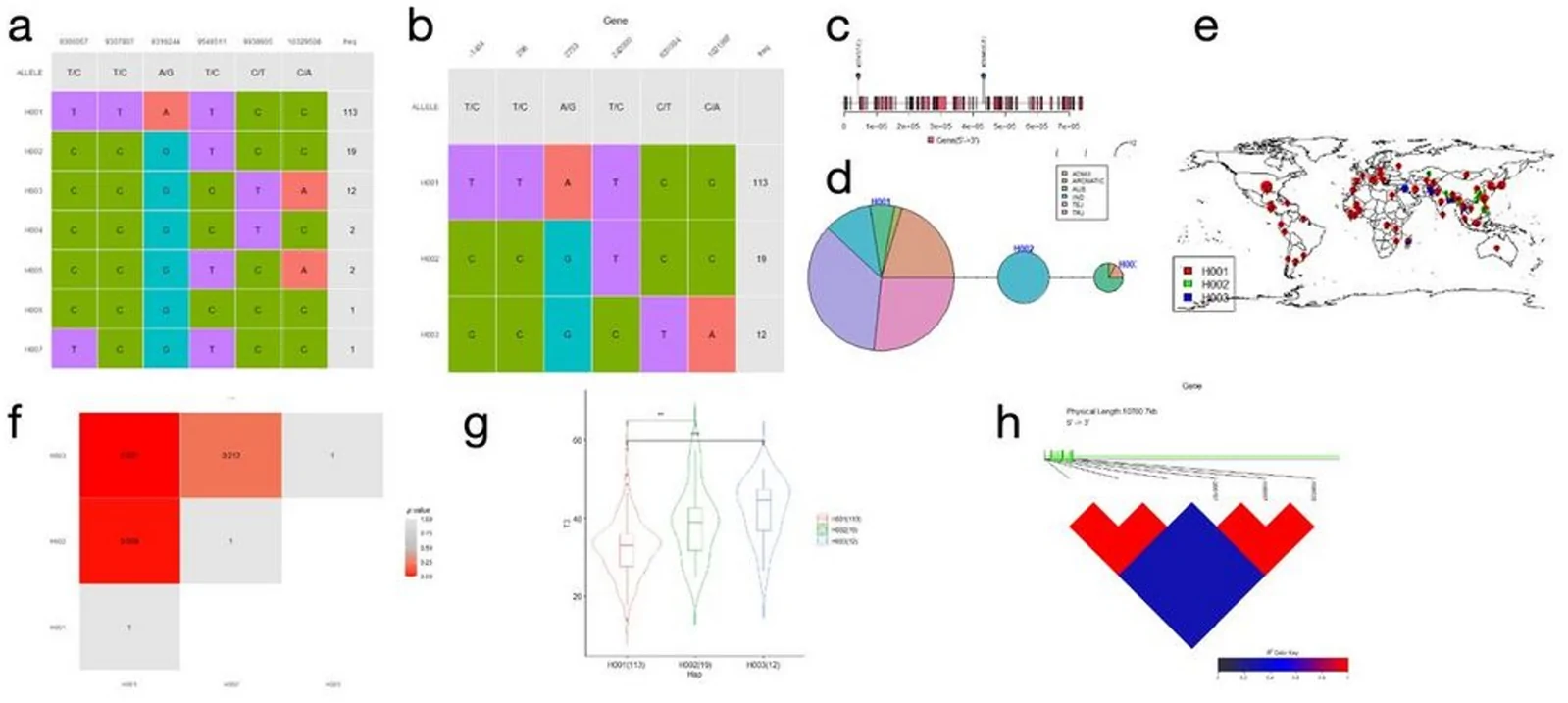
Analysis in qTH31 region for throne height (TH, T3) located on chromosome 3. **a** The first seven haplogroups were observed in 153 accessions using six SNPs: colored boxes are illustrative of haplotype variants with the corresponding frequency in the last column. **significant at *P* ≤ 0.01. **b** Three haplogroups had more than 5 accessions, and gene positions shown in up box. **c** Gene models associated with qTH31: visualization of variant positions above the gene model. The black line represents the genome and rectangles represent upstream and downstream variants. **d** Heatmap for phenotype differences in haplogroups. **e** Violin plot shows the comparison of SBL among accessions between the tree haplotypes of qTH31. **f** Geo-distribution of major haplotypes of qTH31; circle size represents accession counts, and the pies in different colors represent constituent ratios of classified haplotype categories for relevant accessions derived from different eco-regions. **g** qTH31 haplotype network. Each circle represents a haplotype, and the size indicates the accession number. The pies in different colors represent the ratio of categories in each haplotype. The symbol on the line between haplotypes represents the number of variants. **h** LD-block visualization of each site in the locus. The LD block and its red color indicate the close relationship between the SNPs of this haplotype block. The LD-block color key lies at the bottom

Haplotype analysis of TH (thorn height) showed three significant haplotypes in the genomic region qTH31 on rice chromosome 3, with H001 present in 113 accessions, H002 present in 19 accessions, and H003 present in 12 accessions. Haplotype H001 mostly contains accessions belonging to TEJ, TRJ, ADMIX, and IND; haplotype H002 contains just IND; and Haplotype H003 contains AUS, ADMIX, and AROMATIC. Furthermore, three haplotypes show significant differences in trait values (Fig. 4).

### Epistasis analysis

**Fig. 5:**
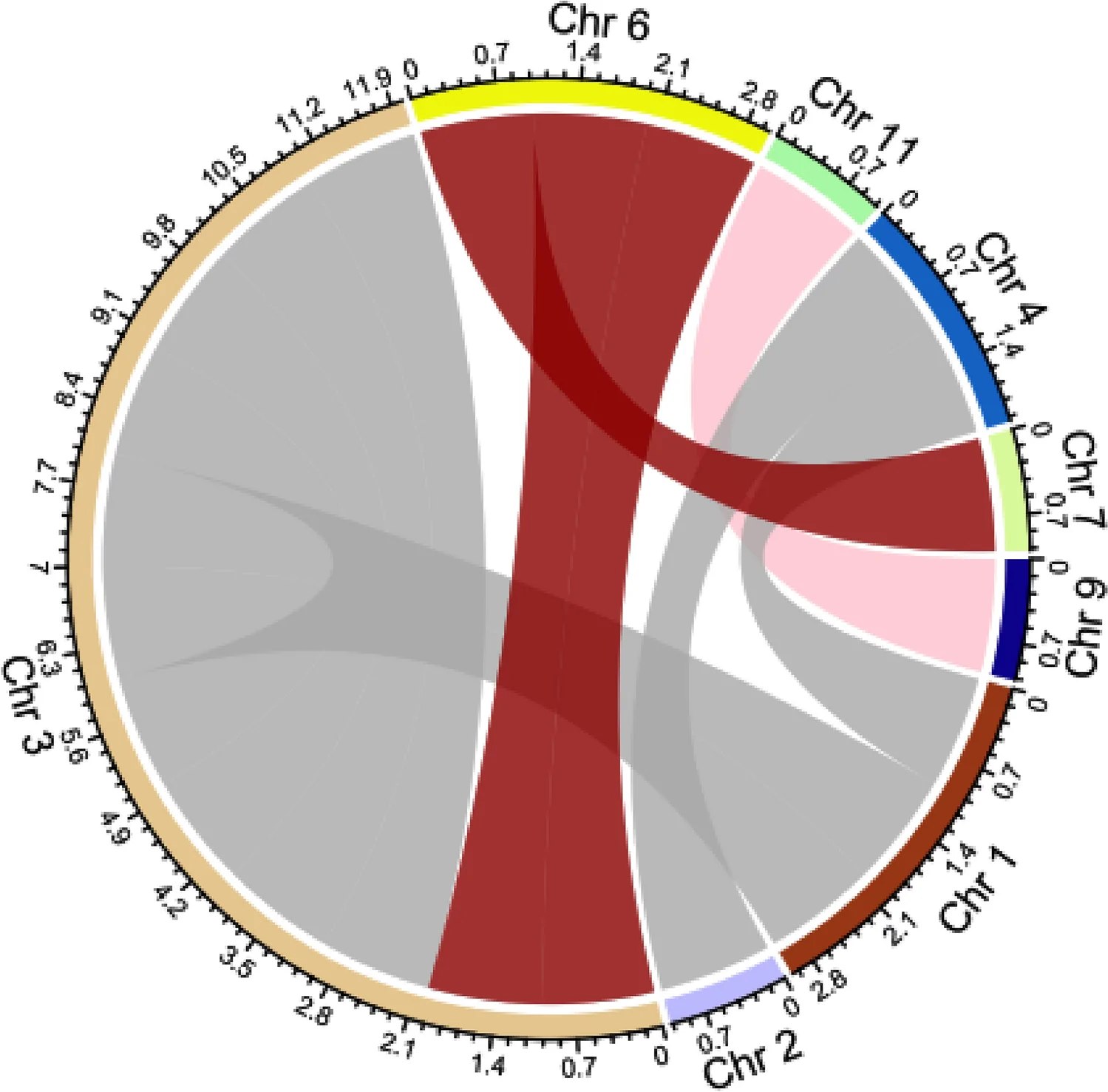
Circus plot of epistatic interactions between chromosomes. Colored crescents around the circle represent different chromosomes (yellow, light green, dark blue, blue, dark red, purple, light brown). The internal area of the circus plot depicts significant epistatic interactions between QTLs affecting silica traits. The pink line is the interaction between two QTLs (SNPs) related to SBW, and the dark gray lines are the interactions between eight SNP (QTL) pairs related to TH. Dark red lines are the interactions between three SNP (QTL) pairs related to TB

The epistasis analyses between our candidate genes identified 18 significant SNP–SNP interactions (cytochrome P450, WD40, zinc finger, DUF247, NB-ARC domain containing protein, pentatricopeptide repeat domain containing protein, heat shock protein Hsp70 family protein, peptidase, ankyrin repeat containing protein, serine/threonine protein kinase, WRKY transcription factor, protein kinase and others) for the SBW, TH and TB traits (Table S10). A circus plot visualizing the epistatic interactions is shown in Fig. 5.

## Discussion

Silicification in rice husks has a significant role in plant structure and mechanical properties. The biogenic intrafibrillar silicification process leads to the accumulation of silica on cellulose nanofibers, which influences silica-particle growth and positively affects the production of different silica types. This process is required for the formation of plant opals, intracellular silicon deposits that contribute to the physical strength and durability of rice husks. Silinite derived from RH ash is an additive that promotes rice crop growth and development. Silicification of rice husks is essential to improve the structural integrity and functional properties of husks (Lux et al. 2020; Nakamura et al. 2021; Satbaev et al. 2021). The presence of silica in rice is associated with multiple biotic and abiotic stress regulation mechanisms. This study explored this variation in a collection of diverse *O. sativa* accessions (153 genotypes collected from across the world). Three GWAS models, i.e., FarmCPU, GLM and MLM, were tested, and FarmCPU was selected for identifying SNPs significantly associated with the traits of interest. Significant SNPs (*n *= 48) were identified, residing in 23 QTLs, and a total of 1027 protein-coding genes were identified as candidates for all traits.

Previously, a rice-straw silica-content study using GWAS resulted to the identification of ATP-binding cassette (ABC) transporters, Casparian thickening, multi-drug and toxin extrusion protein, F-box, and MYB transcription factor as the candidate genes (Gowda et al. 2023). Another rice GWAS suggested that nitrate transporters, ABC transporters, cinnamoyl CoA reductase, *OsCYP*s, and *ONAC*s were new genes associated with morphological changes and relative silicon content (Tran et al. 2024). In our study, *CYP450*s were found flanking significant SNPs for the analyzed traits. Furthermore, genes such as ABC transporter (Song et al. 2014; Hinrichs et al. 2017), F-box protein (Frouin et al. 2019; Gowda et al. 2023), and MYB transcription factor (Wang et al. 2017a, b; Chen et al. 2021; Xu et al. 2023; Roy et al. 2024) were reported for silica content, similar to our findings. Similar to our study, other previously reported genes associated with maize husk traits include chromatin remodeling, oxysterol binding protein-related protein, and GDSL/SGNH-like esterase family (Cui et al. 2016).

### The role of heavy metal detoxifiers and transporters

An *ABC transporter* was found in the flanking region of SBL and TH in this study. Earlier reports indicate the essential role of the ABC transporter family in Si, Cd, or As translocation in plants. *OsABCG25* has a critical role in the formation of the Casparian bands in the exodermis of rice promoted by Si application (Hinrichs et al. 2017; Tran et al. 2024).

BTB domain-containing protein (*BTB* gene) is in the flanking region of TB and SC. The *qSSi3.2* and *OsTAZ24* encode BTB domain-containing genes. The gene promotes plant resistance to various heavy metals, including As and Si (Shalmani et al. 2021), and may help define RH silica content.

A heavy metal transport/detoxification protein domain-containing protein is located in the flanking region of TH. Plant root systems absorb Si from the soil. Consequently, Si is translocated from root to shoot (Khan et al. 2021).

Ankyrin repeat domain-containing proteins are located in the flanking region of SBL and TH. Proteomics analysis revealed new biomineralization proteins of Si. Three of these, coined *dAnk1-3*, contain a common protein–protein interaction domain (ankyrin repeats), indicating a role in coordinating assembly of the silica biomineralization machinery. Knocking out individual *dAnk* genes leads to aberrations in silica biogenesis (Heintze et al. 2022).

### The role of genes involved in growth and development

Zinc finger protein (*Loc_Os06g44750*) is in the flanking regions of an SNP associated with all five traits. It has been reported to be involved in trichome initiation in *Arabidopsis thaliana* (Zhang et al. 2020; Liu et al. 2024). RH contains trichome-like structures, known as pubescence. This similarity led us to consider a similar role for *Loc_Os06g44750* in initiating silica-containing pubescence. Another gene that might be involved in rice trichome development with assumed silica content is coding for homeodomain-like containing protein. This gene is in the flanking region of SBW, SBL, TH, and SC traits. Two distinct trichome types are reported in monocots: macro-hairs and micro-hairs. Macro-hairs are developed on silica cells over the thin vascular bundles, while micro-hairs form along the stomatal cells or adjacent to motor cells (Zhang et al. 2012). Genetic analysis has identified different loci, including glabrous leaf and hull 1 (*gl1*) (Li et al. 2012; Zhang et al. 2012), that are involved in trichome formation in rice (http://www.shigen.nig.ac.jp/rice/oryzabase/; Nagao et al. 1960). *Gl1* encodes a putative protein with an N-terminal homeodomain (HD) and a C-terminal WUSCHEL box motif (WUS), and belongs to the WUS-type homeobox (*WOX*) gene subfamily (Zhang et al. 2012).

The esterase SGNH hydrolase-type domain-containing protein is in the flanking region of an SNP associated with SBL. Leaf morphology is one of the most important agronomic traits in rice breeding because it contributes to crop yield. The drooping leaf (*dr*) mutant was developed from the Ilpum rice cultivar by ethyl methane sulfonate (EMS) mutagenesis. Compared with the wild type, *dr* plants exhibited drooping leaves accompanied by a small midrib, short panicle, and reduced plant height. The phenotype of the dr plant was caused by a mutation within a single recessive gene on chromosome 2, *dr* (*LOC_Os02g15230*), which encodes a GDSL esterase. The analysis of wild-type and dr sequences revealed that the dr allele carried a single nucleotide substitution, glycine to aspartic acid. RNAi targeted to *LOC_Os02g15230* produced the same phenotypes as the *dr* mutation, confirming *LOC_Os02g15230* as the dr gene. Microscopic observations and plant nutrient analysis of SiO_2_ revealed that silica was less abundant in *dr* leaves than in WT leaves (Yu et al. 2020). This study suggests that the dr gene regulates silica deposition and that disruption of silica processes leads to drooping leaf phenotypes.

The *glycosyltransferase* gene is in the flanking region of an SNP associated with TH. Mixed-linkage glucan (MLG), a polysaccharide specific to Poaceae cell walls, and Si have a direct interaction. It is not clear whether MLG is directly chemically linked to either the silica body or to any other form of Si in the rice cell wall. Because MLG is rapidly degraded in the primary cell walls of certain cell types, such as parenchyma, during leaf blade development, silica is unlikely to remain bound to MLG after the leaf stops expanding. In contrast, in the secondary cell walls of cortical sclerenchyma, MLG remains undegraded even after the leaf stops expanding. Loss-of-function mutations in the *CslF6* gene, which encodes a glycosyltransferase capable of mediating the synthesis of MLG, also cause stunted growth, defects in the mechanical properties of mature stems and a disease-lesion-mimic phenotype in leaves (Vega-Sánchez et al. 2012).

### The role of transcription factors

A MYB transcription factor was found flanking SNPs associated with TH and TB. MYB domains are transcription factors that have previously been reported to be involved in Si regulation (Chen et al. 2021; Xu et al. 2023; Roy et al. 2024). Gowda et al. (2023) detected two genes encoding MYB transcription factors. MYB-related transcription factors have previously been shown to be involved in the regulation of expression of certain genes involved in heavy metal (Cd) and metalloid (As) detoxification and/or resistance/tolerance (Gao et al. 2023; Wang et al. 2023). Transcription factor family members mediate Si-mediated As and Cd stress responses by regulating transporter gene expression through GA or ABA signals, among other functions (Lin et al. 2018; Biswas et al. 2023). Additionally, a wheat transcriptome study found that MYB factor genes are differentially expressed when the seedlings are treated with Si (Hao et al. 2021).

TCP domain-containing protein is in the flanking region of an SNP associated with SC. Si could regulate the expression of transcription factor genes (*leDREB-1*, *leDREB-2*, and *leDREB-3*), genes associated with antioxidant enzymes (*leAPX*, *leSOD* and *leCAT* genes), and Si transporter genes (*leLsi-1*, *leLsi-2*, and *leLsi-3*), as well as regulating hormone levels and antioxidative enzyme activities to alleviate the damage caused by salinity stress (Muneer and Jeong 2015).

### The role of stress response genes

Several studies have reported the role of Si in disease resistance by activating defense-related activities in enzymes such as chitinase (Safari et al. 2012; Palacıoğlu. 2024), peroxidases (Yu et al. 2010; Gulzar et al. 2021), polyphenoloxidases (Yu et al. 2010), β−1,3-glucanase (Deng et al. 2020), phenylalanine ammonia-lyase (Yu et al. 2010; Wang et al. 2026), superoxide dismutase (Yu et al. 2010; Gulzar et al. 2021), ascorbate peroxidase (Njenga et al. 2017; Gulzar et al. 2021), glutathione reductase (Gulzar et al. 2021), catalase (Gulzar et al. 2021), lipoxygenase (Gulzar et al. 2021), and glucanase (Safari et al. 2012) which are essential for plant disease resistance responses.

An F-box protein was found in the flanking region of an SNP associated with SBW, TH, TB, and SC. Gowda et al. (2023) detected 13 cyclin-like F-box domain-containing proteins, possibly linked to Si regulation. Twelve of the proteins were found in a single QTL, qSSi10, while one was found in qSSi2. Cyclin-like F-box domains are protein–protein interaction mediators, the genes of which were upregulated upon external Si amendment in response to rice blast stress (Brunings et al. 2009). Also, 12 F-box domain proteins were found in the As QTL regions in flag leaf and dehulled grains (Frouin et al. 2019). This indicates the putative role of F-box domain proteins in Si and As regulation.

Phenylalanine ammonia-lyase (PAL) is in the flanking region of an SNP associated with SBW. Si nutrition not only increases the lignin content but also enhances the activities of peroxides, polyphenol oxidase, and PAL in rice leaves (Zhang et al. 2013).

NB-ARC domain-containing protein is in the flanking region of an SNP associated with SBW and TH. One gene highly expressed in the high-leaf-Si group is predicted to be a disease-resistance gene, with moderate similarity to a probable disease-resistance protein from *O. europaea* and an NB-ARC domain-containing protein when restricted to *A. thaliana* proteins (El-Shetehy et al. 2021). This shows the relevance of silica in disease resistance, with a possibly similar function in RH.

Thylakoid membrane phosphoprotein (*TMP*) is in the flanking region of an SNP associated with SBL and TH. Si can help maintain uneven swelling, disintegration, and missing thylakoid membranes caused by stress (Wang et al. 2019). Furthermore, several photosynthesis-related genes were also regulated by Si supplementation.

### Important haplotypes

A haplotype is a physical grouping of genomic polymorphisms that tend to be inherited together. A specific haplotype typically reflects a unique combination of variants that reside near each other on a chromosome (https://www.genome.gov/genetics-glossary/haplotype). Here, surface block sizes on RH (silica block length and throne height) showed morphological differences among rice varieties. A significant difference was observed in RH throne height between the three haplotype groups in qTH31. The qTH31 haplotype group 1 contains genotypes of TEJ, TRJ, ADMIX, IND; haplotype group 2 contains genotypes of IND; and haplotype group 3 contains genotypes of AUS, Aromatic, and slightly ADMIX. The throne height of the husk in groups 3 and 2 was significantly larger than in group 1. It can be concluded that AUS, AROMATIC and IND genotypes have significantly higher thrones than the other groups. In two haplotype analyses of qSBL91, there was no significant difference in the length of the blocks on RH between the two haplotype groups, but in terms of block length, haplogroup 1 contains mostly TEJ, IND, AUS and ADMIX genotypes, and haplogroup 2 contains mostly TRJ genotypes and insignificant amounts of ADMIX. Although block length did not differ significantly, group 1 has a longer block than group 2.

### The role of epistatic interactions

Significant advances have been made in understanding and predicting genetic interactions that affect quantitative traits. Epistasis accounts for cryptic genetic variation for quantitative traits in natural populations (Mackay 2014). Epistasis describes how gene interactions can affect phenotypes (Miko 2008), an interaction of alleles at different loci, and is a form of non-additive gene action (Holland 2001). Assessing the effects of pairwise and higher-order epistatic interactions between polymorphic DNA variants on molecular interaction networks is of great importance (Mackay 2014). A study on silica cells in rice showed that cell wall lignification occurs before silica deposition and that silica body formation is dependent on a pre-formed cell wall matrix (Zhang et al. 2013). It was reported that lignin extracted from rice can induce silica deposition and act as a template for silica formation (Fang and Ma 2006). Since laccase enzymes are responsible for lignin polymerization, this gene family is considered one of the strongest candidates for silicification (Zexer et al. 2023). Cytochrome P450 genes are involved in the synthesis of monolignols (lignin precursors (Zexer et al. 2023). Silica deposition is correlated with specific lignin properties. Laccase and cytochrome P450 are likely to be a powerful biological module for studying silica genetics (Zexer et al. 2023). Glycosyl hydrolases rearrange wall polysaccharides, acyltransferases modify wall phenolics, and laccases form lignin. These three groups are placed in a common biological network related to silicification (Zhang et al. 2019). In silicon-related transcriptomic studies, different transcription factor families, including Zinc finger and bHLH, have been repeatedly reported as differentially expressed genes. These genes are likely regulators of cell wall pathways and the response to silicon, but there is no direct evidence for their role in phytolith formation (Zexer et al. 2023).

## Conclusion

The study identified QTLs and candidate genes related to rice husk silica content and morphology in *O. sativa*, which will help explain the genetic basis of these traits across a panel of *O*. *sativa* accessions. A literature review showed that some of the genes in the vicinity of GWAS signals, genes flanking significant SNPs, have already been reported as relevant to silica content in rice. In this study, we identified the most important markers associated with rice husk silica traits on chromosomes 2, 6, and 9. The candidate genes associated with these traits are involved in growth and development, heavy metal detoxification and transport, stress response, and transcriptional regulation. Our results improve understanding of the genes that define silica content in RH.

## Supplementary Information

Below is the link to the electronic supplementary material.

Supplementary file1 (DOCX 9289 KB)

Supplementary file2 (XLSX 93 KB)

## Data Availability

The genotype data are available from public resources. The phenotypic data are available from the authors upon request.

## Abbreviations

GWAS: Genome-wide association study
QTL: Quantitative trait locus
RH: Rice husk
SNP: Single nucleotide polymorphism
