# Supplementary material for: Dissecting the genetic basis of rice husk silica content and morphology using GWAS
Source: Planta. 2026 Oct 22;264(6):174. doi: 10.1007/s00425-026-05211-8 (PMC13690876; doi:10.1007/s00425-026-05211-8)
Supplement: Supplementary file 1 [file 425_2026_5211_MOESM1_ESM.docx]

**Supplementary figures**

|  |  |  |
| --- | --- | --- |
|  |  |  |
| Fig. S1 Trait distributions. Silica block width (SBW, T1), Silica block length (SBL, T2), Thorn height (TH, T3), Thron base (TB, T4), Silica content (SC, T5). | | |

**Fig. S2** Principal component (PC) biplot and population structure from MVP package in R. The plot shows three main subpopulations.

**Fig. S3** Phylogenetic tree of a kinship plot that efficiently separates the 153 accessions in five main geographical clusters of subpopulations (TEJ; temperate japonica. IND; india. AUS; aus. ARO; aromatic. TRJ; tropical japonica). A heatmap of the values in the kinship matri, showing the level of relatedness among the population, this analysis drawn by popkin and gplots.

|  |  |  |
| --- | --- | --- |
|  |  |  |
|  |  |  |
|  |  |  |
| **Fig. S4** LD decay on 12 chromosomes of rice. pink Points indicating LD between pairs of markers, R2 values of LD are plotted as a function of the distance between pairs of markers. The black line showed the regression used to describe the relationship between the pairwise distance and R2 (0.4666 for all chromosomes with different distances for chromosomes in Supplementary Table 3), we generated this analysis with Tassel and qqplot2.. | | |

**Fig. S5** The figure shows the distribution of SNPs on chromosomes. We drawed this in MVP package(R). Red and orange boxes are parts with full of SNP on chromosomes.

|   **a** |   **b** |
| --- | --- |
|   **c** |   **d** |
|   **e** |   **f** |
|   **g** |   **h** |
| Fig. S6 analysis in qSBL91 region for SBL (silica block length, T2) located on Chromosome 9. a) at first eleven haplogroups were observed in 153 accessions using nine SNPs: colored boxes are illustrative of haplotype variants with the corresponding frequency at the last column. **significant at p ≤ 0.01. b) two haplogroups hade more than 5 accestions and we showed gene positions in up box. c) gene models associated with qSBL91: visualization of variants position above gene model, the black line represents the genome and rectangles represent upstream and downstream variants. d) heatmap for phenotype differents in haplogroups showed in d fig. e) violin plot shows the comparison of SBW among accessions between the two haplotypes of qSBL91. f) geo-distribution of major haplotypes of qSBL91; circle size represent accessions counts and the pies in different color represent constituent ratios of classified haplotype categories for relevant accessions derived from different eco-regions. g) qSBL91 haplotype network. Each circle represents a haplotype and the size indicates accession number. The pies in different color represent the ratio of category in each haplotype. The symbol on line between haplotype represents number of variants. h) LD-block visualization of each site in the locus. LD-block and its red color indicate the close relationship between the SNPs of this haplotype block. The LD-block with colors key lie at the bottom. | |
